# Factors That Affect Return to Sports After an Achilles Tendon Rupture: A Qualitative Content Analysis

**DOI:** 10.1177/23259671221145199

**Published:** 2023-02-10

**Authors:** Unnur Sædís Jónsdóttir, Annelie Brorsson, Katarina Nilsson Helander, Roy Tranberg, Maria E.H. Larson

**Affiliations:** † Department of Orthopaedics, Institute of Clinical Sciences at Sahlgrenska Academy, University of Gothenburg, Gothenburg, Sweden.; ‡ Department of Clinical Neuroscience and Rehabilitation/Physiotherapy, Institute of Neuroscience and Physiology, Sahlgrenska Academy, University of Gothenburg, Gothenburg, Sweden.; § Primary Health Care, Region Västra, Götaland, Sweden.; *Investigation performed at Institute of Clinical Sciences at Sahlgrenska Academy, Gothenburg, Sweden*

**Keywords:** achilles tendon, rehabilitation, psychological aspects of sports, return to sports, qualitative research

## Abstract

**Background:**

Achilles tendon rupture is common among physically active individuals, yet a high percentage fail to return to their former activity after the injury. Quantifiable factors such as type of treatment, hours of rehabilitation, and age have not been associated with return-to-play rates. A factor that influences recovery is the participant’s experience before and throughout the rehabilitation process, which can be explored using a qualitative content analysis.

**Purpose:**

To explore and describe what influences the participant to return to physical activity after an Achilles tendon rupture.

**Study Design:**

Cross-sectional study; Level of evidence, 3.

**Methods:**

Twenty participants (14 men; mean age, 46 years) were interviewed as part of this study. All participants had ruptured their Achilles tendon 4 to 6 years before the interviews. From the interviews, codes were extracted that evolved into 19 subcategories, 6 categories, and 1 theme.

**Results:**

The overarching theme that emerged was “Help me and then I can fix this.” The 6 categories were (1) one’s own drive to succeed, (2) having a supportive social network, (3) trusting the support from the health and social systems, (4) receiving and adapting information from others drives persistence in returning to activity, (5) impact of the injury on psychological factors; and (6) influence of physiological aspects.

**Conclusion:**

To be able to recover properly from an Achilles tendon rupture and return to activity, the study participants described the importance of obtaining the support needed to be able to gain optimal rehabilitation. In the participants’ opinion, for a greater chance of successful treatment and rehabilitation, it was vital to be provided with good support.

An Achilles tendon rupture (ATR) is a common injury among physically active individuals, particularly in middle-aged men performing recreational sports.^6,14^ The incidence of ATR in Sweden has been increasing through the period of 2001 to 2012 (male: 47-55.2/100,000 person-years; female: 12-14.7/100,000 person-years).^14^ A systematic review and meta-analysis was published regarding the rate and measurements of returning to play after an ATR.^35^ On average, approximately 80% of those who rupture their Achilles tendon return to their previous activity after rehabilitation, with large variability: 28% to 100% return to activity/sports/play.^20,21,35^

A great deal of effort has been put into finding the optimal treatment after an ATR. Many systematic reviews and meta-analyses have been published, without reaching consensus on which treatment method is preferable: surgical or nonsurgical.^9,27,28,34,36^ Recent research has concluded that if there is access to well-performed functional rehabilitation, a nonsurgical method may be preferable, as there is an increased risk of infection with surgical treatment.^34,36^ Another systematic review^9^ concluded that the risk of rerupture was lower for those who had surgical treatment, but there was no difference in deep venous thrombosis, return to sports, or range of motion. A comparison between groups using 2 patient-reported outcome measures (PROMs), the Achilles tendon Total Rupture Score (ATRS) and Physical Activity Scale (PAS), did not reveal any differences either.^9^ Ochen et al^23^ stated that "the final decision on the management of acute ATRs should be based on participant-specific factors and shared decision making."^23^ From these reviews, it is apparent that there are pros and cons to both treatments.

It has also been shown that early weightbearing and accelerated rehabilitation have a positive effect on functional outcomes after surgical treatment of ATR.^4,8^ A systematic review was performed that found 12 studies comparing early rehabilitation with immobilization.^4^ They divided the early rehabilitation into 3 categories: full weightbearing, early ankle mobilization, and a combination of the 2. All the categories showed better results compared with immobilization, and the combined treatment had the highest satisfaction level. However, the results after early rehabilitation after nonsurgical treatment were not as significant. Early weightbearing did not show any significant difference in terms of the ATRS when looking at endurance and strength using the heel-rise work test, rate of reruptures, or return to work and sport; however, in terms of health-related quality of life, there was a significant difference in favor of the early weightbearing group.^2^

Other important aspects of optimal recovery after an ATR are the individual’s experiences of the treatment and rehabilitation process as well as one’s intrinsic factors. To evaluate a part of these aspects, PROMs have often been used, but to acquire a deeper understanding of the individual’s experience, a qualitative research method may be more appropriate. This has previously been implemented with regard to the factors that influenced a participant to return to sports/play after an Achilles tendon repair,^25^ an anterior cruciate ligament reconstruction,^5,31^ hip arthroscopy for femoroacetabular impingement,^29^ and arthroscopic Bankart repair.^30^ Peterson et al^25^ performed a semistructured qualitative assessment in which 23 individuals were interviewed 2 to 7 years after their surgically treated ATR. Of those 23, 6 had returned to their preinjury level of sport. From these interviews, 3 themes were identified: personal motivation, shift in focus, and confidence in health care team. They concluded that many athletes accept not returning to their previous level of sport but are satisfied with their recovery anyway.

The aim of this study was to use a qualitative content analysis research approach to explore and describe what influences the participant’s process during treatment and rehabilitation to increase our understanding of factors affecting return to sports after an acute ATR.

## Methods

### Recruitment of Participants

The study protocol was approved by the regional ethics review board, and all participants provided written informed consent. All the participants came from a previously performed study^16^ in which the aim was to evaluate the difference in lower leg biomechanics between those who were afraid and those who were not afraid of reinjury at 2 years after an acute ATR. The previous study comprised 30 participants. The data collection in the previous study was performed in 2016 at the Department of Orthopaedics, Institute of Clinical Sciences at Sahlgrenska Academy, Gothenburg, Sweden. The inclusion criteria in the original study were age 18 to 65 years, having a closed midsection rupture diagnosed and treated either with or without open surgery initiated within 4 days of injury, proficiency in the Swedish language, and no other injuries or diseases that would preclude one from performing the functional tasks involved.

**Figure 1.**
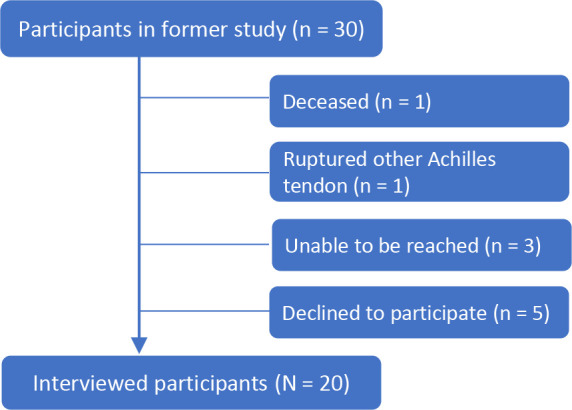
Flowchart of participant inclusion.

In the present study, data were collected in 2019. One goal was to obtain participants with large variations in age, sex, treatment, function, symptoms, and physical activity after the injury. In the original study, 25 participants had answered PROMs regarding function, symptoms, and physical activity using the ATRS^22^ and PAS^12^ at 2 years after the injury. In the present study, 20 of these participants were included (Figure 1). They had also answered the question regarding their physical activity compared with the level of activity before the injury. Answers were given on a 5-level scale (1 = a lot less active; 5 = a lot more active). It was possible to include participants from all levels of activity. The participants also answered the following yes/no question: "Do you ever refrain from any activity due to the fear of reinjuring your Achilles tendon?"

### Data Collection

Participants were contacted 4 to 6 years after the injury by telephone by one of the authors (A.B.) to inquire whether they were willing and able to participate in the present study. They were given a short description by telephone of the goal of the study and the research topic, and they were assured that their participation was voluntary and that they could withdraw at any time. The interviewer and first author (U.S.J.) met the participants for the first time at the time of the interviews.

One of the authors (U.S.J.), a woman, an experienced physical therapist with a Master of Science degree, and a doctorate student at the time of writing, conducted the semistructured interviews and was alone with the participants during the interviews. This was the first time she had taken part in a qualitative study.

Nineteen interviews were performed in a conference room at the Centre for Orthopaedic Research at Sahlgrenska Academy. One interview was performed in a quiet room at a physical therapy clinic in Gothenburg. The participants were given oral and written information when they arrived at the interview and were also given time to ask any questions.

Each interview took between 15 and 40 minutes to complete, and each participant was interviewed once. The interviews for this study were completed in April and May of 2019. A study-specific guide was used to ask open-ended questions, with the aim of not interrupting the participant unless the discussion moved a long way from the aim of the interview. The interviewer took notes to be able to ask relevant questions but relied on the records and transcription when performing the analyses. The interviews were audio recorded and transcribed by an impartial secretary. The participants did not read the transcripts at any time and did not provide feedback on the results. In the Results section, direct quotations from the participants are indicated by italicized text.

### Data Analysis

An alphanumeric identifier was used to preserve the anonymity of each participant. A thematic qualitative content analysis was used to narrate the answers obtained from the participants, identifying meaning units, codes, subcategories, categories, and a theme. Computer software, NVivo Version 12 (QSR International Pty), was used to analyze the data. No predefined definitions were used in the analysis. Responses were grouped hierarchically according to a 2004 study by Graneheim and Lundman^11^ on qualitative content analysis in nursing research. In their study, the authors describe how the overarching theme is found from categories put together from codes that are derived from meaning units from the text.

All the interviews were performed by the same individual (U.S.J.), who also extracted meaning units and initial coding. The analysis was performed with guidance from one of the coauthors (M.E.H.L.), who is an experienced researcher in this method. Two of the interviews were coded by all authors and an additional 2 by U.S.J. and M.E.H.L. The first author coded the remaining 16 interviews. Codes were derived from the text, and they were kept close to the original text. They were then categorized into 19 subcategories. Furthermore, in the process of reaching a consensus between the authors, the subcategories were discussed and evaluated by all the authors. The subcategories were then abstracted into 6 categories, from which 1 theme emerged.

## Results

The demographics of the 20 study participants are presented in Table 1. Six categories emerged from the qualitative content analysis of the 20 interviews. The results are outlined in Table 2, where categories, subcategories, and example codes are put forward.

**Table 1:** Demographics of the Participants at the Time of Interviews (N = 20)*^a^*

Variable	Value
Age, y	
Mean ± SD	46.2 ± 9.76
Median (range)	47 (27-62)
Sex, male/female, n	14/6
Time from rupture, mo	
Mean ± SD	60.3 ± 8.3
Median (range)	63 (49-71)
Body mass index	
Mean ± SD	26.0 ± 2.7
Median (range)	26.4 (21.4-30.6)
ATRS*^b^* (n = 18)	
Mean ± SD	80.3 ± 13.9
Median (range)	84.5 (56-100)
PAS*^b^* (n = 18)	
Mean ± SD	3.9 ± 1.3
Median (range)	4 (2-6)
Physical activity compared with before injury*^b^* (n = 18)	
Mean ± SD	2.3 ± 1.0
Median (range)	2 (1-5)
Fear of reinjury, yes/no, n*^b^* (n = 18)	9/9
Treatment type, surgical/nonsurgical, n	9/11

*^a^* ATRS, Achilles tendon Total Rupture Score; PAS, Physical Activity Scale.

*^b^* Responses were collected during the original study^16^ and were conducted 2 years after the initial injury (2016).

**Table 2:** Categories, Subcategories, and Examples of Codes From Qualitative Content Analysis.

Category	Subcategory	Code
1. One’s own drive to succeed	A. Importance of having something to work toward (goal) B. Importance of a positive mentality C. Motivating to be a part of research	a. Important to have a reason for the exercises b. Fatal if you think it is boring c. Dared to do more after participating in the research
2. Having a supportive social network	A. Flexibility at work during the rehabilitation period B. Getting help from friends and family C. Receiving encouragement from others	a. Had great understanding and help from my supervisor b. Stayed with parents when he had the plaster on c. Would have been lazier without pressure from the wife
3. Trusting the support from health and social systems	A. Experience that the health care workers were professional, caring, and able to convey knowledge and security B. Ambiguity about who and what makes the decision on treatment C. Well-adapted rehabilitation pushed progress D. Effect of personal economy on this period E. Accessibility or availability to be mobile.	a. Got inspiration from the physical therapist b. Thought it was strange to be asked which treatment I wanted c. Got exercises to do at home, not just at the rehabilitation clinic d. Couldn’t afford a car or a taxi, had to cycle e. Would not have been able to take the bus, so I needed help with transport
4. Receiving and adapting information from others drives persistence in returning to activity	A. Experience of others who have ruptured their Achilles tendon to promote self- improvement	a. Athletes inspired; if they can do it, so can I
5. Impact of the injury on psychological factors	A. Acceptance and adaptation to the situation B. Fear of reinjury C. Perception of psychological limitations in activities of daily living	a. Having had previous injuries, helped adapting b. Was afraid of becoming disabled again c. Felt really hopeless
6. Influence of physiological aspects	A. How physical fitness before the injury affected the process B. Own physical fitness after the injury apart from the Achilles tendon C. Perception of physiological change after Achilles tendon in activities of daily living D. How pain obstructed the rehabilitation process	a. Had always exercised; that helped to understand how important rehabilitation is b. It is not because of the calf muscle that I don’t run normally c. The Achilles tendon feels better now, after the injury d. Had some pain when I had overused the tendon during rehabilitation; otherwise, there was no pain

### Category 1: One’s Own Drive to Succeed

When facing a project such as recovery after an injury, the desire to show progress is crucial. This category therefore describes the importance of having something to work toward after an injury and how important it is to be mentally positive throughout the period. It also outlined how motivational it is to be a part of research.

#### Importance of Having Something to Work Toward (Goal)

It was crucial for the participant to return to activity after an injury, so the focus shifted toward that goal. To reach the goal, the participants found it was important to be consistent in rehabilitation and follow the instructions from health care workers with determination.

*So, I did my rehabilitation, because I wanted to be back.…I wanted to be able to do sports with full intensity again. (participant 9* *)*

The participants described how they understood the importance of the exercises for recovery and were willing to work toward their goals. The commitment to continue to spend time following the rehabilitation program made the reward (goal) attainable.

#### Importance of a Positive Mentality

Mentality can both be a limitation and a drive to continue progress when working toward recovery.

*When a longer time had passed, I became lazier. But all the prerequisites were there. (participant 15)*

There was nothing to prevent the execution of the rehabilitation, but the drive and discipline to follow the instructions were lacking. On the other hand, their own determination and personal character could help to maintain focus and discipline with their eyes on the goal.

*I used to be in the army; as a soldier, I learnt to do things. To be disciplined when helping myself. (participant 8)*

Taken together, it is good to have a positive mentality when working through the rehabilitation phase.

#### Motivating to Be a Part of Research

Insecurity about one’s own ability after ATR can be a factor that limits progress. To reduce that insecurity, a challenge such as taking part in a study exploring physical performance increased courage.

*After I took part in the study and did all the tests, I felt that I dared to push my limits. I’ve played soccer, done ice skating, played golf, and I do everything I want to do. (participant 7)*

One’s own eagerness to be physically active was identified with participation in the study (the previously mentioned study the participants took part in 2 years after the injury). Showing the examiner in the research, their ability increased their determination to attain better physical health.

*It was very cool to be encouraged to participate [in the study].…I wanted to show that I was better. (participant 13)*

Therefore, the physical therapists’ recognition of the participants’ progress made the individual more determined.

### Category 2: Having a Supportive Social Network

The support from family, friends, and coworkers was described as vital when encountering obstacles that their own physical strength and mental state were not capable of overcoming. Getting help from friends and family was important when the participants experienced helplessness, as well as receiving encouragement in going through the rehabilitation process. Flexibility at work during the rehabilitation period can also be a key to successful recovery.

#### Getting Help From Friends and Family

The support needed to be able to return to activity can come in many forms, such as grocery shopping, cleaning, or even carrying a coffee cup through a door, as well as encouragement to perform exercises.

*It actually worked out, and I also had very nice friends that helped me with grocery shopping and picking up things. That kind of help made everything easier. (participant 15)*

Help in any form reduced the mental load of everyday living.

#### Receiving Encouragement From Others

Being asked to take part in activities can be encouraging and assure one that one’s ability is still there.

*It was really nice to know that they wanted you to take part. That you are good enough. It is…motivating. (participant 15)*

This encouragement could help the participants challenge themselves to take the next steps toward activity. Family was important when the injured individual was reminded to keep up with exercises.

*When he was at home, my partner asked, “Have you done your exercises?” to remind me of them. Even my family asked how the exercises were going. (participant 10)*

Encouragement from friends and family can give participants an extra impulse when continuing with the rehabilitation.

#### Flexibility at Work During the Rehabilitation

Flexibility at the workplace when feeling disabled after an injury can also be a relief, and the understanding of a manager and colleagues can mean a lot to an employee.

*Actually, I was able to get a customized job description. When I was jumping around with crutches, I didn’t meet any patients. I was just working as an administrator.…I got a lot of understanding from colleagues…and I had a very understanding supervisor. (participant 3)*

The support from a manager in the form of helping the participant to be able to work could make the participant more comfortable in one’s work environment and, as a result, able to ask for further adjustments. Flexibility at work was also important.

*I have a very flexible job. So, I could just say that I would be away [for training]. It is not as if I have to register at a certain time, no one stands there waiting. (participant 16)*

This kind of flexibility helped the participants with their rehabilitation, leading toward increased strength and endurance, which resulted in a better chance of being active.

### Category 3: Trusting the Support From Health and Social Systems

This category describes how critical it is that the participant feels that the health care workers are professional, caring, and able to convey knowledge and security. In the first hours or days after the injury, it seemed like there was ambiguity about who and what makes the treatment decisions. But later on, well-adapted rehabilitation pushed progress. The effect of personal economy on this period should also be considered, as the reduction in work was often unavoidable for recovery. Another reason for reducing work could be difficulties with transportation; therefore, accessibility or availability to be mobile is crucial.

#### Experience That the Health Care Workers Are Professional, Caring, and Able to Convey Knowledge and Security

Instructions from those who have the education and experience to guide the participant toward favorable results were usually trusted the most. Many people can give advice, but in the end, it is those the participant trusts who have the greatest impact. When the health care professionals acted insecurely and did not know how to take care of injured participants, the participants felt that they were not getting the best treatment possible and were therefore hesitant about the process.

*I thought it was very good to be at the physiotherapy clinic. They were very capable. It felt like the right thing, in my opinion, to be in an environment where people know what they are doing, they understand and have allocated time for this. It felt right. (participant 17)*

The feeling that one was secure and in good hands helped the process when situations were out of one’s control.

#### Ambiguity About Who and What Makes the Treatment Decisions

There was uncertainty among the participants regarding which treatment should be used initially, as there was no fixed protocol for which of the 2 treatments, surgical or nonsurgical, should be chosen. Making the decision regarding surgery depended on the doctor who was taking care of the participant, and sometimes the participants were offered the chance to make the decision themselves. This could be confusing for the participant.

*I remember from my visit to the emergency room that they asked me if I wanted an operation or not. I thought it was a strange question, as they should know what is best. (participant 18)*

Then again, the participant might prefer to be able to choose.

*Now, later, I wonder if they didn’t think I was worth the operation because they put a cast on. I was probably too old for the operation, I don’t know. I would have chosen the operation, I think. (participant 4)*

The participants found it difficult to understand why some were surgically treated and others not. That might question their own significance to the doctor making the decision.

#### Well-Adapted Rehabilitation Pushed Progress

Receiving and following a rehabilitation program that the participants trusted and seemed sensible pushed the progress. The guidelines had to be clear, and individual focus was essential.

*I always followed the guidelines. Otherwise, there was a risk that I would push the limits too far. But I didn’t dare with this injury, so I followed the guidelines all the time and felt how I became better and better. (participant 2)*

The participants expressed increased motivation when they felt that the work they were putting into the rehabilitation paid off and their strength was increasing as it should. They felt assured that their decisions were right.

#### Effect of Personal Economy on This Period

Depending on the job, it can be difficult to work during the first weeks after the injury. In injuries like an ATR, it was important to rest with the foot up high for the first few weeks, but that might not have been possible when the participant was required to work in an office or do physical work. While one preferred to be at home, it was not always possible because of practical and economic issues.

*And then it was the thing with the economy. I didn’t want to take sick leave because I was able to work. I didn’t meet any new patients because I had to use my body a lot for that. But I attended meetings and so I felt that I could work. (participant 20)*

Income was reduced, as the sick-leave pay was lower than the participant’s normal wages.

#### Accessibility or Availability to be Mobile

It was important to have good access to transportation for easy access to the workplace and rehabilitation. Using a cast and a brace for the first 6 to 8 weeks could limit one’s ability to walk a long distance. Difficulties with transportation and subsequent increased activity could increase pain and make the recovery slower.

*It has been simple and convenient for me to do my exercises at a gym that is close to my home. It is easy for me to go to the gym, and at the time, I drove a car all the time. (participant 18)*

The location of the gym/clinic was important, as not being able to walk, take public transport, or drive long distances could affect the time available for training.

### Category 4: Receiving and Adapting Information From Others Drives Persistence in Returning to Activity

This category describes how receiving information about the injury can guide one through the experience. Today, where communicating with people from all over the world is relatively easy and we can read stories about others’ rehabilitation over the internet, it is possible to use the experience of others to promote self-improvement.

#### Experience of Others to Promote Self-Improvement

People who had the same injury were often willing to share their experience, good or bad. It was described as encouraging to listen to others who had the same injury and had recovered successfully. “Role models” can be important for recovery and motivation. Hearing about those who have not done so well can also be an important learning opportunity.

*I had also heard about those who did not do well.…This person did not do the rehabilitation as recommended and was still limping a few years later. This sparked even more motivation for me. (participant 9)*

Others’ experiences can shift the focus toward the right path with guidance and encouragement.

*My mood went down a little, but then I read about others that had had the same injury. Then I changed my focus and started focusing on rehabilitation instead. (participant 11)*

The participants said that when they realized others were dealing with the same problem as they were, it did not seem as overwhelming.

### Category 5: Impact of the Injury on Psychological Factors

This category outlines the challenges participants face psychologically when dealing with an ATR. Acceptance and adaptation to the situation can be vital when dealing with the consequences of an ATR. The reaction of the mind to the body, such as fear of reinjury and perception of psychological limitations in activities of daily living, may have an impact on movement ability.

#### Acceptance and Adaptation to the Situation

Mental acceptance that the injury had occurred accelerated the adaption to the circumstances. The participants described how preparing the mind for a long rehabilitation process and being accustomed to the disability after an injury could help acceptance. Previous experience can accelerate adaptation with learning effects from previous injuries.

*I had broken the foot in which I ruptured the Achilles tendon many years ago.…But I think it really helped. (participant 17)*

Some factors helped in retrospect, but at the time it was happening, the help was not clear.

*Often, I wanted to stay at home. I think the fact that I live a long way from work helped, as it forced me to cycle with my crutches on the handlebars. (participant 11)*

Being in a difficult situation, but at the same time being forced to adapt, can be meaningful in retrospect.

#### Fear of Reinjury

Not trusting the healing process and being afraid of reinjury may delay the rehabilitation process, thereby affecting the return to activity.

*I run close to my summer cottage, because it is better for my feet to run off road. But no floorball, no tennis, no soccer, no handball, no more ATRs. My thought is that I won’t take a chance on something like this. (participant 18)*

This means that fear of reinjury might discourage participants from activities they used to perform as they associate them with their injury.

#### Perception of Psychological Limitations in Activities of Daily Living

An ATR can be limiting in many respects, as the participants felt unable to perform the simplest tasks because of the tendon, and they even felt helpless at times. Another aspect could be that there were no limitations but still an unconscious avoidance of certain circumstances that could affect activities of daily living.

*I can do everything I want. There is no disability that I feel, but I cannot go for hikes, and what I have noticed, because we have a sailing boat, is that I am not secure when walking on deck. It is not the same. (participant 8)*

Insecurity affected the choices that were made, but it did not always stop the performance.

### Category 6: Influence of Physiological Aspects

The participants described other physical impairments that were affected during and after the injury, which might have influenced rehabilitation. This is described in the subcategory of physical fitness before the injury can affect the process. Physical fitness after the injury could also vary with increased age, as well as with other injuries affecting fitness. The physiological change after an ATR in activities of daily living can cause problems affecting physical ability and even pain that can obstruct the rehabilitation process.

#### Physical Fitness Before the Injury Can Affect the Process

Finding time to perform the rehabilitation could often be a challenge, but if the daily routine before the injury included exercises, it could be easier to adjust the rehabilitation program to that routine.

*I went to the gym before the injury. All the prerequisites were there for me. And maybe it was also that I was training before it happened and my routine was therefore there, so I went and trained. (participant 18)*

Adding the rehabilitation to a fitness program can be less of a challenge than adding the gym to a daily routine.

#### Physical Fitness After the Injury

Apart from the injured tendon, other factors can affect physical fitness. Even though the tendon has healed as well as possible, hip pain, for example, can be a limiting factor in achieving what was hoped for.

*I cannot run, I cannot run the Gothenburg half marathon anymore, cannot run 10 or 20 km. I can run 5 km nowadays. That is how much I can run without getting pain in my hip. (participant 8)*

#### Physiological Change After an ATR in Activities of Daily Living

There are other factors that can be limiting for return to activity after an ATR. The injury might have caused weakness in the injured foot and affected the movement pattern during activity.

*I am impaired. I really am. I am still weaker on the injured side, and I sometimes experience a small problem with my spine, because I am placing the load incorrectly when I run. (participant 16)*

After a serious injury, the healing could be very good, but not perfect. There can be a change in skin sensation, the connection to the muscle, or a feeling of stiffness because of scar tissue.

*I feel less strength in my calf muscle, reduced feeling, less contact with the muscle, some exercises don’t work as well as before, in the gym that is. It also affects my work, as it is more difficult to stand on a ladder. (participant 1)*

This physiological difference after the injury is said to affect activities in daily living and the performance of certain activities.

#### Pain Obstructing the Rehabilitation Process

These physiological changes might have slowed the rehabilitation process if they caused pain due to excessive exercise and not enough rest.

*There were 2 incidents [during rehabilitation] when I told myself that I need to take it slowly and rest for a week or so, or actually do fewer exercises. (participant 7)*

Adjusting the rehabilitation to physical symptoms and understanding why the pain was there could help with adaptation during the rehabilitation process.

## Discussion

The findings illustrate 2 parts of an overarching theme: “Help me and then I can fix this.” On one hand, there is the importance of obtaining help and support from the people around you, and on the other hand, there is one’s own ability to follow the progress needed for improved function. However, getting help and executing the rehabilitation program are not the only factors affecting return to activity. Even though the participants performed the rehabilitation protocol perfectly, the tendon had been damaged, and scar tissue cannot replace the original tendon.^1^ With support and encouragement from friends, family, and the physical therapist, strength and endurance were restored to the greatest extent possible. The role of friends and family was important in making life easier during the first weeks after the injury, especially when the ability to manage activities of daily living was reduced. The participants described how a push from the physical therapist would make them realize that they were capable of more than they thought and that their trust in the physical therapist was crucial when challenging the strength of the tendon. This would increase their belief in themselves and their own potential. With restored self-efficacy in the ability of the tendon/muscle, half the battle is won. The other half is having the determination and focus when following instructions. The participants described how their own mentality helped them maintain focus on the process, but also that, with the previously mentioned encouragement, the execution of the exercises was easier to remember for those lacking focus.

Different personalities can lead to different results, and as described in category 1 (one’s own drive to succeed), this is a vital personality trait for good results. Conti et al^7^ interviewed 10 professional basketball players with regard to returning to their preinjury level. Their results consisted of 2 general dimensions that support our category: coping skills and motivation. Coping skills were important for resilience when performing the rehabilitation, but also when facing stressful situations. Their motivation came from their love of their sport and the importance of full recovery, in order to be able to play as they did before the injury happened.^7^ The participants were not professional athletes, but they did discuss the importance of working toward a goal, which was to return to their sport. Only 1 of the participants scored an level of activity as 5, on a scale of 1 to 5, compared with preinjury level. On average, it was 2.3 for the group, which can be interpreted as indicating that the return to preinjury level for the participants in the present study was not very successful.

Category 2 describes how support of the participants’ own social network (family, friends, and coworkers) is important, as they encourage the participants to maintain their focus on recovery. They also mentioned small things such as being asked how things are going or offering help with transportation/shopping. Participants’ acknowledgment of their disability, and their willingness to take the time to make their day a bit easier, is important. Johnston and Carroll^15^ conducted a qualitative study in which they evaluated different types of social support to injured athletes. The importance of emotional and practical support, provided by friends and family, decreased with time, as the guidance of physical therapists and coaches increased.^15^ This agrees with the responses from the participants in the present study, who mentioned how helpful it was to have support when walking on crutches and needing help with activities of daily living.

Several participants mentioned the importance of feeling secure, that the doctors and physical therapists knew what they were doing and saying. Category 3 described the feeling of trusting the support from the health and social systems. It was confusing for participants when 2 treatment options were available. The treatment options after an ATR are nonsurgical or surgical, and so far, the optimal treatment has not been identified with regard to complications such as reruptures and infection rates.^28^ There is a trend toward treating athletes more often with surgery, and some participants explained that if they had been elite athletes, they would have had surgery. The surgical treatment is viewed as superior. Some of the participants who were not operated on mentioned that it appeared as though they had received the inferior treatment, while others said they would have chosen nonsurgical treatment. When there was no ambiguity about the next step and they knew what was required of them, this appeared to motivate and help the process.

In the study by Peterson et al^25^ on return to sports after an Achilles tendon repair, the authors reported that finding the best surgeon and physical therapist one knew of and being willing to commit to the rehabilitation guidelines was important for the healing process. This is in agreement with another study that interviewed patients who had returned to sports, as well as their parents, after an anterior cruciate ligament reconstruction with regard to how they experienced physical therapy during their rehabilitation and how it affected their outcome.^24^ The results were that the relationship between patients and physical therapists was very important, as many patients and their parents mentioned it in the interviews. Subthemes that emerged were the role of the physical therapist as a guide, motivator, booster of confidence, and coordinator of care. The physical therapists’ communication skills were also of great importance, as well as “the ability to make physiotherapy sessions comfortable and fun.”^24^ This is in line with the present study; if one is certain that one has received the correct guidance and feels that all the aspects are being considered, it is easier for the participant to trust the process. Taken together, a clinical guideline could therefore be to provide the patient with adequate, individualized, and—between the different health care providers—consistent information throughout the whole rehabilitation process.

Nowadays, where unlimited information is only a click or a phone call away, comparisons with others in the same situation are easy. There may be many benefits; being able to relate to others that have experienced something similar is important, as well as obtaining information related to the rehabilitation process. The downside can be that horror stories on the internet and unrealistic victory stories can be easy to access and are often more prominent than the “normal” ones. Taken together, receiving and adapting information from others can drive persistence in returning to activity was category 4, which described how inaccurate information can produce unrealistic goals of the process. Unfortunately, despite searching various databases, we did not find any studies supporting or contradicting the speculation regarding the impact of outside information on recovery after musculoskeletal injury, but we found a study regarding a dermatological condition. Pithadia et al^26^ conducted a cross-sectional study looking at YouTube videos as a source of patient information, and there were 199 sources that met the inclusion criteria. Natural treatments accounted for the majority of the videos (55.3%), with 36.6% being A and B recommendations from the American Academy of Dermatology, while 7.0% discussed a mix of all the treatments. The majority (56.3%) of the videos were financially biased. They concluded that the majority of the videos discouraged seeking medical advice and that dermatologists should consider posting evidence-based information on YouTube.^26^ When reviewing what the participants said, they often had heard/read stories that both increased their motivation to follow the guidelines from the physical therapist and calmed them down when they realized how common the injury was. Others found the difference in treatment confusing, whereas the difference in treatment protocols between parts of the country was obvious. Our understanding is that patients should be careful when collecting information from people who are not health care workers, working with their kind of injury, as information on the internet can be presented from a nonmedical perspective, as in the Pithadia et al study.

Category 5, related to fear of reinjury and insecurity that the tendon was not able to perform as it should, was often mentioned as the impact of psychological factors on the injury and return to activity. The importance of this can be seen in another study that showed how physical improvements were inferior when returning to sports after injury and self-reported function was reduced for those who showed signs of fear of reinjury compared with those who were not afraid.^13^ The avoidance of certain activities was mentioned, as the participants were stressed that the tendon might be reinjured, were insecure/did not trust the calf/Achilles tendon, and were unable to perform the required task. This avoidance can be connected to a model presented in 2000 by Vlaeyen and Linton^32^ and redesigned in 2007 by Leeuw et al^18^ as “the Fear-Avoidance Model of Musculoskeletal Pain,” in which the consequences of an injury were explained. When sustaining an injury, pain is usually experienced, but what happens then can move into 2 directions, either toward less fear, confrontation, and then recovery or toward catastrophizing, moving to fear of pain, and escape/avoidance, which can lead to disuse/disability/depression. This can then lead to more pain and the patient’s reentering the circle of the model.^18^ Some of the participants went in the second direction and therefore avoided using the muscle/tendon, leading to disuse, after which they possibly experienced reduced strength and endurance in the muscle/tendon.

Many of the participants described category 6, the influence of physiological aspects, as limitations in the Achilles tendon or calf muscle in the form of stiffness, less endurance, and/or less strength. A recent study about return to sports and patient satisfaction after nonsurgical treatment for an acute ATR presented quantified results, where 94% (of 89 participants) were satisfied with the treatment, even though only 70% had returned to their preinjury sport at the 1-year follow-up and 73% at 5 years.^19^ At the 1-year follow-up, the participants had 84% strength on the injured side compared with the uninjured side, measured with the single-leg heel-rise test.^19^ Self-evaluation of physical ability was measured using the ATRS questionnaire and PAS that compared present activity level with the preinjury level. The ATRS examines limitations during various activities such as running and jumping. Table 1 shows the results of the ATRS, and it is obvious that there were large variations between participants. The mean score was 80.3 (of 100 = no limitations), with a range of 56 to 100. The comparison between before and after the injury was evaluated using a scale from 1 to 5 (1 = a lot less active, 3 = equal to before, 5 = a lot more active). The mean value was 2.3, which can be interpreted as indicating that most of the participants were less active than before the injury. Accordingly, the results present somewhat less activity compared with before the injury.

### Strengths and Limitations

In qualitative studies, there is a tradition of discussing the importance of the trustworthiness of a study, which indicates comprehension of the methodology of the study and that the reader has reason to trust the researchers who are conducting the study.^10^ Trustworthiness consists of credibility, dependability, transferability, and authenticity. For good credibility, it is important to recruit participants who may have the experience being studied and are able to express their experience, while a limitation would be recall bias. In this study, 4 to 6 years had passed since the injury, which might be considered a long time, as recall bias could be prominent. But as deficits have been recorded 6 and 7 years after the injury,^3,33^ it is likely that any deficits were still there for our participants. The credibility can also be reflected in the appropriate number of participants and the variability between them. According to Graneheim et al,^10^ there is no predetermined number of participants for any study, but Kvale and Brinkmann^17^ recommended a sufficient number of participants to be able to transfer every possible aspect. In a standard interview study, the interview tends to comprise about 15 ± 10, and the present study of 20 participants is therefore within the recommended limits.^17^ This number of participants should increase transferability; that is, the number should reflect the potential for the results to be transferred to other contexts.

Dependability refers to the analysis of the text, where codes are derived from the transcript and are supported by quotes from the original text. They are then abstracted to subcategories, categories, and an emerging theme. The analysis can vary between researchers, as their own experience and understanding are different. These factors can affect the interviewer’s follow-up questions and interpretation of the answers.^10^ The first author of this study conducted 2 pilot interviews, transcribed them, and analyzed them under the guidance of the most experienced coauthor with regard to the qualitative content analysis research form. Those interviews and analyses were not used in the study. Furthermore, 4 interviews were analyzed to reach consensus regarding the codes used for further analysis.

These levels of abstraction and interpretation, from quotes, to codes, to subcategories, to categories, to theme, produce the risk of reduced authentication, thereby reducing trustworthiness.^10^ We therefore put examples of codes, subcategories, and categories in a table (see Table 2) for readers to interpret whether the authentication was sufficient.

In this study, participants were recreational athletes who performed their sports/activity in a completely different environment from professional athletes. This could be both a strength, as most athletes who injure their Achilles tendon are recreational, and a limitation, since it would be interesting to be able to evaluate professional athletes as well.

## Conclusion

This study identifies 1 main theme, “Help me and then I can fix this,” which emphasizes that, first, the patient needs help/support when an injury occurs and is then able to help oneself. The help/support comes from various directions, but it can be equally important. If this help is lacking, there can be obstacles on the road to recovery that can lead to difficulty performing the necessary rehabilitation, together with challenges returning to activity. With the help available, important factors stopping people from returning to activity are the lack of focus and positive mentality. The focus and positive mentality are also supported by the people around one and, with those factors combined, the rest is less of a challenge.
